# Complete Genome Sequence of a Porcine Parvovirus Strain Isolated in Central China

**DOI:** 10.1128/genomeA.01247-13

**Published:** 2014-01-30

**Authors:** Lin-Qing Wang, Yan Wang, Long-Biao Chen, Peng-Fei Fu, Hong-Ying Chen, Bao-An Cui

**Affiliations:** a College of Animal Science and Veterinary Medicine, Henan Agricultural University, Zhengzhou, Henan Province, China; b Molecular Biology Laboratory, Zhengzhou Normal University, Zhengzhou, Henan Province, China

## Abstract

We report here the complete genome sequence of the porcine parvovirus (PPV) strain J-PPV, isolated from central China. Our data, together with sequence data for PPV isolates from other regions of China, will help in understanding the epidemiology and molecular characteristics of PPV field isolates in China.

## GENOME ANNOUNCEMENT

Porcine parvovirus (PPV) is a negative-oriented single-stranded DNA virus belonging to the genus *Parvovirus* and the family *Parvoviridae*; it is the major causative virus in a reproductive failure syndrome in swine characterized by infertility, mummified fetuses, early embryonic death, and stillbirths (1). The viral genome is a single-strand DNA approximately 5 kb in length, with a terminal palindromic structure. The PPV particle is composed of three viral polypeptides, VP1, VP2, and VP3, with molecular masses of 83, 64, and 62 kDa, respectively (2).

Since the first occurrence of PPV infection in China was reported in 1982, several PPV strains have been isolated from many Chinese provinces (3, 4). To date, the complete genome sequences of several PPV strains from China are available in GenBank. To investigate the epidemiological and molecular characteristics of PPV isolates in China, we sequenced the complete genome of PPV strain J-PPV, which was isolated from fetuses with suspected PPV infection on a pig farm in Henan province, central China.

Six pairs of oligonucleotide primers were designed according to the PPV genome sequences. The PCR products were cloned into the pMD18-T vector (TaKaRa Biotechnology, Dalian, China) and sequenced with an Applied Biosystems (ABI) 3730xl DNA analyzer. The whole genome of the J-PPV strain is 5,075 bp in length, with a G+C content of 37.91%, and consists of a 291-bp 5′ noncoding region (NCR), a 526-bp 3′ NCR, and a 4,258-bp coding region. The PPV genome includes two open reading frames (ORFs) (5); ORF1 is located at the 5′ end and ORF2 is located at the 3′ end. The ORF1 gene encodes the nonstructural (NS) proteins NS1 (1,989 bp in length, 662 amino acids [aa]), NS2 (486 bp in length, 161 aa), and NS3 (324 bp in length, 107 aa); ORF2 encodes the structural proteins VP1 (2,190 bp in length, 729 aa), VP2 (1, 740 bp in length, 579 aa), and a late nonstructural protein, SAT (207 bp in length, 68 aa).

J-PPV shows 95.6% to 99.8% nucleotide sequence identity to previously reported PPV strains, 99.8% with the attenuated PPV strain NADL-2 (GenBank accession no. NC_001718) and 95.6% with the virulent PPV strain Kresse. The NADL-2 strain differs from the Kresse strain by 6 amino acids in the VP2 protein, which have been demonstrated to be responsible for the replication efficiency of PPV in cell culture (6). Four of the 6 amino acids are the same between J-PPV and NADL-2, 1 amino acid is the same between J-PPV and Kresse, and the remaining amino acid is different in the three strains. In addition, both J-PPV and Kresse differ from NADL-2 by four other amino acids in other locations. The phylogenetic tree of the genome nucleotide sequences was constructed using the MEGA 5.1 software (7). J-PPV was nearest to the NADL-2 strain and farthest from the Nanjing200801 strain. These results revealed that J-PPV has the characteristics of virulent and vaccine PPV strains. These data will be helpful for analyses of the evolutionary epidemiology and molecular pathogenesis of PPV.

### Nucleotide sequence accession number.

The GenBank accession no. for J-PPV is KF742500.
